# Prognostic significance of the systemic immune‐inflammation index in pancreatic carcinoma patients: a meta-analysis

**DOI:** 10.1042/BSR20204401

**Published:** 2021-08-02

**Authors:** Xiaocheng Li, Huapeng Lin, Renbin Ouyang, Yaowei Yang, Jing Peng

**Affiliations:** 1 Department of Hepatobiliary Surgery, The First Affiliated Hospital of Hunan University of Medicine, Huaihua, Hunan, P.R. China; 2 Department of Intensive Care Unit, Affiliated Hangzhou First People's Hospital, Zhejiang University School of Medicine, Hangzhou, Zhejiang, P.R. China; 3 Department of General Surgery, University-Town Hospital of Chongqing Medical University, Chongqing, P.R. China

**Keywords:** meta-analysis, pancreatic carcinoma, prognosis, systemic immune-inflammation index

## Abstract

**Background:** Systemic immune-inflammation index (SII) is a prognostic indicator for several malignancies, including pancreatic carcinoma; however, there is no consensus on its significance. In the current study, a systematic meta-analysis was used to explore the correlation between SII and prognosis in pancreatic carcinoma patients.

**Methods:** PubMed, Embase and Cochrane Library databases were screened from inception to May 2020. Studies describing the prognostic role of SII in pancreatic carcinoma were then retrieved. The pooled hazard ratio (HR) and 95% confidence interval (CI) was calculated using random- or fixed-effects models to determine the correlation between SII and prognosis.

**Results:** A total of four studies, comprising 1749 patients, met the inclusion criteria of the study and were therefore included in this meta-analysis. The meta-analysis showed that high SII indicated was correlated with worse overall survival (OS) in patients with pancreatic carcinoma (HR: 1.43, 95% CI: 1.24–1.65, *P*<0.001). These findings were validated through subgroup analyses, stratified by the American Joint Committee on Cancer (AJCC) stage. In addition, patients with high SII showed poorer cancer-specific survival (HR: 2.32, 95% CI: 1.55–3.48, *P*<0.001). However, analysis showed no significant correlations between SII and disease-free and relapse-free survival (RFS).

**Conclusion:** These findings indicate that SII is a potential non-invasive and a promising tool for predicting clinical outcomes of pancreatic carcinoma patients. However, the current research did not explore whether neoadjuvant therapy has an effect on the prognostic value of SII. Further studies using adequate designs and larger sample sizes are required to validate these findings.

## Introduction

Pancreatic carcinoma is the seventh leading cause of cancer-related mortalities in the world. In addition, it is one of the most devastating gastrointestinal tumors with a 5-year survival rate of approximately 9% [1,2]. Despite advances in approaches for early diagnosis and therapy, no significant improvement has been achieved in survival benefits of pancreatic carcinoma patients, owing to distal metastases or local recurrence [3]. Therefore, it is important to develop novel and specific prognostic biomarkers for risk stratification in pancreatic carcinoma patients.

Recent studies report that inflammatory response is highly correlated with tumorigenesis, development and metastasis of various tumors [4–7]. For example, hematological biomarkers, such as levels of lymphocytes, neutrophils, platelets, neutrophil to lymphocyte ratio (NLR), platelet to lymphocyte ratio (PLR) and C-reactive protein, are currently commonly determined and applied in assessment of systemic inflammatory response [8,9]. Previous studies report that these parameters are potential prognostic markers in patients with solid tumors [10–13]. Numerous studies have also implicated systemic immune-inflammation index (SII), combining platelet, lymphocyte and neutrophil counts, as a promising inflammation‐based biomarker and a prognostic indicator for clinical outcomes in several malignancies [10,14–19], including pancreatic carcinoma. However, some studies report inconsistent or contradictory results on the prognostic value of SII [20,21]. Therefore, the current study sought to comprehensively explore the clinical prognostic effect of SII in pancreatic carcinoma patients through a meta-analysis.

## Methods

### Search strategy and study selection

The present study was registered at PROSPERO (number CRD42021241231) and was performed following the PRISMA guidelines for meta-analysis [22]. Two investigators (Xiaocheng Li and Huapeng Lin) independently performed a systematic online literature search and data extraction. Electronic scientific databases, including PubMed, Embase and Cochrane Library, were searched and relevant studies published from inception up to May 2020 retrieved. Search terms used included ‘systemic immune-inflammation index’, ‘SII’, ‘pancreatic neoplasms’, ‘pancreatic’ and ‘pancreas’, and all searches conducted using a combination of MeSH terms and free-test words. Furthermore, references of relevant systematic reviews and included studies were retrieved manually and additional studies identified.

### Inclusion and exclusion criteria

Inclusion criteria included studies that: (1) described the relationship between SII and prognosis of patients with pancreatic tumors; (2) reported no laboratory or clinical evidence of infection, autoimmune or blood disease; (3) included an optimal cut-off value of SII; (4) reported outcomes of interest, including cancer-specific survival (CSS), overall survival (OS), disease-free survival (DFS) or relapse-free survival (RFS); (5) in which the hazard ratios (HRs) with 95% confidence intervals (CIs) of prognostic factors could be extracted or calculated; (6) were written in English language. On the other hand, exclusion criteria were as follows: (1) studies on cell lines, tissues or animals; (2) studies in which necessary data were not available; (3) studies reporting continuous variables for SII; (4) studies in which the publication type was case series, review article, letter, editorial or commentary.

### Data extraction and quality assessment

All data required for meta-analysis were independently extracted by two authors using a prespecified table, and any disagreements were resolved through discussion to reach a consensus. Extracted information included, name of the first author, country of origin, publication year, study period, sample size, study design, baseline characteristics of patients, follow‐up time, American Joint Committee on Cancer (AJCC) stage, treatment methods, SII cut-off value, outcome, HRs and the corresponding 95% CIs.

HRs and 95% CIs for univariate and multivariate analyses were obtained through two ways. Firstly, HRs and their corresponding 95% CIs were directly obtained from the reported literature. Secondly, in cases where the article only provided the Kaplan–Meier curves, HRs and 95% CIs were calculated from the survival curves using Engauge Digitizer version 4.1 [23]. Methodological quality for each eligible article was independently assessed by the two reviewers using Newcastle–Ottawa Scale (NOS). NOS scores ranging from 1 to 9 points were considered low to high quality [24], with a score ≥6 indicating high quality.

### Statistical analysis

Meta-analysis was conducted using RevMan software, version 5.3 (The Nordic Cochrane Center, Cochrane Collaboration and Copenhagen, Denmark). Summary statistics were performed using standard meta-analysis methods, with HRs used as an effect measure to assess association between SII and prognosis in pancreatic carcinoma patients. Between-study statistical heterogeneity was determined using Higgins *I*^2^ statistic and Cochran’s Q test, with data followed by *P*<0.10 and/or *I*^2^ > 50% considered to have significant heterogeneity. In cases where studies showed a significant homogeneity, a fixed-effects model was adopted, otherwise a random-effects model was used. Presence of publication bias was determined using funnel plot. Stability and reliability of the results were determined using sensitivity analysis through the leave-one-out strategy. *P*<0.05 were considered statistically significant.

## Results

### Search results and study characteristics

**Figure 1:**
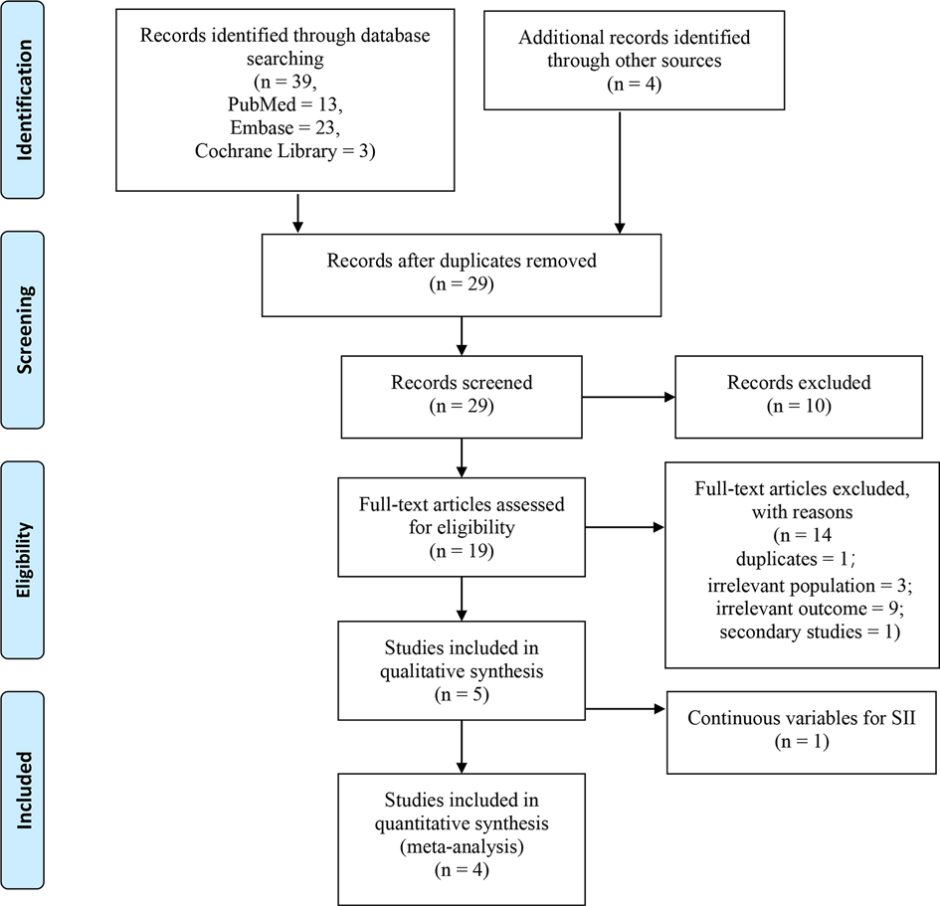
Flow chart showing the study selection process

A total of 43 articles were obtained from the search, including 23, 13, 3 and 4 studies from EMBASE, PubMed, Cochrane Library and other sources, respectively. A total of 19 articles were identified after exclusion of duplicates. Further, 14 articles were excluded after reviewing abstracts and full text as they were systematic reviews, case reports, comments and irrelevant studies. In addition, a study by Zhou et al. [11] was excluded as it reported SII as a continuous variable. A summary of the search procedure is presented in Figure 1. A total of four studies [18,19,21,25], involving 1749 patients were included in the current analysis. Three of the studies reported OS, whereas the other study reported CSS and RFS. One of the studies included two independent patient cohorts, comprising 197 and 222 patients in the training and validation cohorts, respectively [25]. In this study, data from the two independent patient cohorts were extracted as the cases in the two cohorts were independent, and there were no overlapping cases. Therefore, in this meta-analysis, the two patient cohorts were treated as two independent studies in subsequent analysis. The included articles were all retrospective cohort studies and were published between 2018 and 2020. The sample size of included studies ranged between 321 and 590, and SII cut-off values ranged from 440 to 900. All included studies had NOS scores ≥ 6. A detailed description of all included studies is presented in Table 1.

**Table 1:** Characteristics of included trials in the review

Author	Study design	Study period	Number of samples	Age (years)	Treatment	Time point of SII measurement	Tumor type	AJCC stage	Median follow-up (months)	Outcome	Cut‐off value	NOS score
Murthy et al. (2020) [21]	Retrospective study	2007–2017	419	65.17 ± 9.7	Surgery/NAT + Surgery	Before and after NAT	PDAC	I–III	39.1 (32.6–48.1)	OS	900	8
Jomrich et al. (2019) [19]	Retrospective study	1995–2014	321	68.5 (35.9–92.3)	Surgery/NAT + Surgery	Before surgery	PDAC	I–III	60	OS, DFS	873	8
Zhang et al. (2019) [25]	Retrospective study	2011–2015	419	61 (25–84)	Mixed	Before treatment	PA	III–IV	48	OS	440	8
Aziz et al. (2018) [18]	Retrospective study	2004–2015	590	65.5 (35.5–82.4)/65.6 (36.6–84.7)	Surgery/NAT + Surgery	Before surgery	PDAC	I–III	43.3/44.8	CSS, RFS	900	8

Abbreviations: NAT, neoadjuvant therapy; PA, pancreatic adenocarcinoma; PDAC, pancreatic ductal adenocarcinoma.

### Relationship between SII and OS in pancreatic carcinoma patients

**Figure 2:**
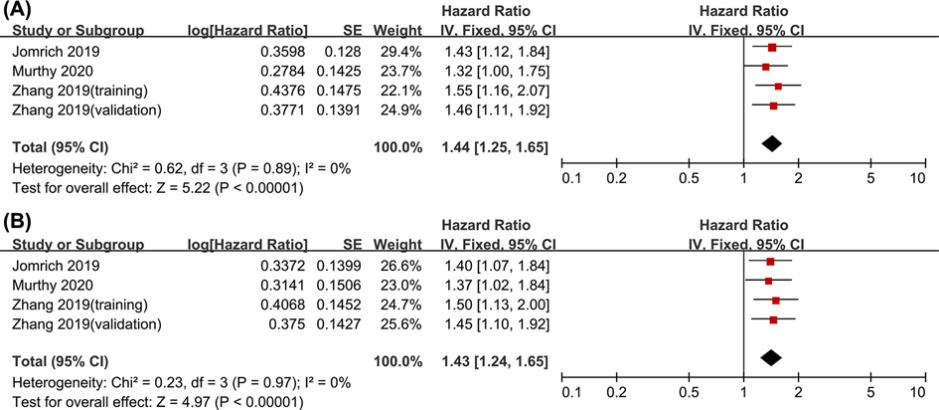
Forest plots showing correlation between SII and OS (**A**) Univariate analysis and (**B**) multivariate analysis.

A total of three cohort studies, comprising 1159 patients, were combined to evaluate the relationship between SII and OS in pancreatic carcinoma patients. Analysis showed no significant heterogeneity among the studies (*I*^2^ = 0), therefore, a fixed-effects model was used for analysis. Univariate analysis showed a pooled HR of 1.44 with a corresponding 95% CI of (1.25–1.65) (*P*<0.001), whereas multivariate analysis showed an HR of 1.43 with a corresponding 95% CI of (1.24–1.65) (*P*<0.001). These findings showed that a higher SII is correlated with poor OS in pancreatic carcinoma (Figure 2).

### Relationship between SII and CSS/DFS/RFS in pancreatic carcinoma patients

Only one study reported the prognostic value of SII on CSS/DFS/RFS in pancreatic carcinoma patients. The findings showed that a high SII is an independent prognostic marker for CSS (HR: 2.32, 95% CI: 1.55–3.48, *P*<0.001); however, no significant differences were observed between SII and DFS (HR: 1.27, 95% CI: 0.95–1.70, *P*=0.103) or RFS (HR: 1.49, 95% CI: 1.00–2.20, *P*=0.048) in pancreatic carcinoma (Table 2).

**Table 2:** Meta and subgroup analyses for SII in pancreatic carcinoma

Analysis	Number of studies	Number of cases	HR (95% CI)	*P*‐value	Heterogeneity	
					*I*^2^ (%)	*P*-value
OS	3	1159	1.43 (1.24–1.65)	<0.001	0	0.97
AJCC stage						
I–III	2	740	1.39 (1.13–1.69)	0.001	0	0.91
III–IV	1	419	1.48 (1.21–1.80)	<0.001	-	-
CSS	1	590	2.32 (1.55–3.48)	<0.001	-	-
DFS	1	321	1.27 (0.95–1.70)	0.103	-	-
RFS	1	590	1.49 (1.00–2.20)	0.048	-	-

### Subgroup and sensitivity analyses

A summary of results from subgroup analyses stratified by AJCC stage is presented in Table 2. A higher SII was a poor prognostic indicator for OS in patients with stage I–III (HR: 1.39, 95% CI: 1.13–1.69, *P*=0.001) and stage III–IV (HR: 1.48, 95% CI: 1.21–1.80, *P*<0.001). Moreover, all subgroups showed significant correlation between SII levels and OS of pancreatic carcinoma (Table 2). Subsequent exclusion of a single trial did not alter pooled HRs for these relationships. A comparison among the random- and fixed-effects models showed an identical pooled risk estimate of OS. Subgroup and sensitivity analyses indicated that the current pooled evidence from meta-analysis was robust and credible.

### Publication bias

**Figure 3:**
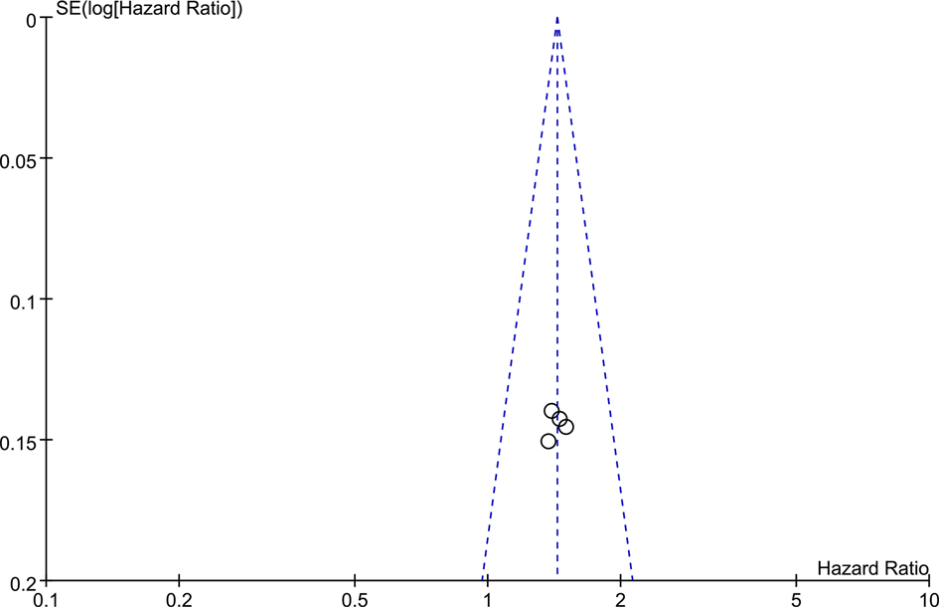
Funnel plots showing correlations between SII and OS

Funnel plots used in the meta-analysis did not show any evidence of publication bias among included studies (Figure 3).

## Discussion

Several studies report that inflammatory response plays an important role in development and progression of tumors [6,26,27]. This interaction is mediated by a series of inflammatory cells, which form major cellular components of the tumor microenvironment [28]. Tumor microenvironment either directly or indirectly affect tumor cell proliferation, migration and angiogenesis by releasing various inflammatory mediators [29,30]. Studies report that peripheral blood inflammatory cell count, including neutrophils, and lymphocytes is implicated in prognosis of several tumors [31–34]. SII is a combined marker based on platelet, lymphocyte and neutrophil counts which was first identified as an independent factor for predicting clinical outcomes of hepatocellular carcinoma patients [14]. Thereafter, its prognostic value was reported in various tumors, including colorectal, renal cell, esophageal squamous cell, lung, prostate and gastrointestinal cancers [15–17,35–37].

SII is a novel and convenient marker, that is easily accessible and inexpensive. Recent studies report that SII is a prognostic marker for pancreatic carcinoma. In the present study, literature review-based analysis of the relationship between SII and prognosis of pancreatic carcinoma patients showed that patients with higher SII levels have worse prognosis, possibly due to several reasons. Firstly, neutrophils promote release of a variety of inflammatory factors, such as vascular epithelial growth factor, neutrophil elastase, interleukin-8 and matrix metalloproteinase-9, which play important roles in promoting invasion, proliferation, progression and metastasis of cancer cells, and help these cells to escape immune surveillance [38,39]. In addition, platelets promote adhesion of tumor cells to microvascular endothelium and form a defense barrier around circulating tumor cells, helping tumor cells in escaping the immune surveillance of the host [40]. Moreover, they directly promote tumor growth and metastasis by secreting pro-angiogenic factors and platelet-derived growth factors [41]. Conversely, lymphocytes play an important role in defending the body against cancer cells by inducing cell death, and through inhibition of cell proliferation and migration [5]. Therefore, a reduction in the number of lymphocytes may weaken antitumor immune response and immune surveillance function, thus providing a good microenvironment for tumor growth [42,43]. High levels of SII, platelets and neutrophils and low levels of lymphocytes, enhances tumor invasion, adhesion, progression, metastasis and weakens antitumor immune response through these mechanisms. Therefore, a high level of SII is associated with adverse clinical outcomes in tumor patients.

In the present study, SII’s prognostic value in pancreatic carcinoma patients was determined based on four published studies comprising 1749 patients. Pooled results indicated that pancreatic cancer patients with high levels of SII had worse OS rates compared with those with low SII levels. Furthermore, subgroup analyses were used to explore the prognostic significance of SII in patients with different stages. The findings showed that high SII level was a potential independent poor prognostic marker for OS in patients with resectable or advanced pancreatic tumors. In addition, a high SII value was associated with CSS in patients with pancreatic tumors, although these results need further validation. Aziz et al. [18] reported that SII is an independent predictor of CSS in patients with resectable pancreatic cancer, regardless of whether the patient had received neoadjuvant therapy or not. However, Murthy et al. [21] recently reported that for patients with resectable pancreatic cancer who had received neoadjuvant therapy, SII before neoadjuvant therapy was not correlated with the patient’s prognosis, and only SII after neoadjuvant therapy was an independent predictor of OS. These findings from the two studies are inconsistent. Therefore, the effect of neoadjuvant therapy on evaluation of the prognostic value of SII should be explored further.

The current study had several limitations. Firstly, the four included articles were retrospective cohort studies, and could have inherent limitations. Secondly, the total number of included studies and the sample size was relatively small, especially for some subgroup analyses. Therefore, more large-sample studies should be conducted to further validate these findings. Thirdly, stratified comparisons based on other factors, such as age, gender and pathological type of tumor were not performed owing to the paucity of the original data from individual studies. Fourthly, the optimal cut-off value of the SII was inconsistent across different studies. Fifthly, the relationship between SII before and after neoadjuvant therapy and prognosis was not assessed at subgroup level due to lack of relevant data. Finally, although the funnel plots suggested no evidence of publication bias, this may be because funnel plots lacked adequate statistical power owing to the small number of studies included. The study findings should be interpreted with caution owing to the several limitations.

In summary, high SII levels are correlated with poor prognosis of OS and CSS in pancreatic carcinoma patients. However, SII is no significantly correlated with DFS and RFS. The exact biological mechanisms underlying the observed correlations have not been fully explored, although previous studies have implicated the inflammatory response pathway in regulation of SII. The current study findings indicate that SII can be used as a non-invasive and valuable prognostic marker for resectable or advanced pancreatic carcinoma in clinical applications. However, the current research did not explore whether neoadjuvant therapy has an effect on the prognostic value of SII. Therefore, well designed, large‐scale and prospective studies should be conducted to evaluate and validate the correlation between SII levels and the prognostic outcomes of pancreatic carcinoma patients.

## Data Availability

All data are available from the authors upon request.

## Abbreviations

AJCC: American Joint Committee on Cancer
CI: confidence interval
CSS: cancer-specific survival
DFS: disease-free survival
HR: hazard ratio
NOS: Newcastle–Ottawa Scale
OS: overall survival
RFS: relapse-free survival
SII: systemic immune-inflammation index
